# *SMAD6*-deficiency in human genetic disorders

**DOI:** 10.1038/s41525-022-00338-5

**Published:** 2022-11-21

**Authors:** Ilse Luyckx, Aline Verstraeten, Marie-José Goumans, Bart Loeys

**Affiliations:** 1 Centre of Medical Genetics, Faculty of Medicine and Health Sciences, University of Antwerp and Antwerp University Hospital, Antwerp, Belgium; 2 Department of Human Genetics, Radboud University Medical Center, Nijmegen, The Netherlands; 3 Department of Cell and Chemical Biology, Leiden University Medical Center, Leiden, The Netherlands

**Keywords:** Genetics research, Molecular medicine

## Abstract

*SMAD6* encodes an intracellular inhibitor of the bone morphogenetic protein (BMP) signalling pathway. Until now, SMAD6-deficiency has been associated with three distinctive human congenital conditions, i.e., congenital heart diseases, including left ventricular obstruction and conotruncal defects, craniosynostosis and radioulnar synostosis. Intriguingly, a similar spectrum of heterozygous loss-of-function variants has been reported to cause these clinically distinct disorders without a genotype–phenotype correlation. Even identical nucleotide changes have been described in patients with either a cardiovascular phenotype, craniosynostosis or radioulnar synostosis. These findings suggest that the primary pathogenic variant alone cannot explain the resultant patient phenotype. In this review, we summarise clinical and (patho)genetic (dis)similarities between these three *SMAD6*-related conditions, compare published *Madh6* mouse models, in which the importance and impact of the genetic background with respect to the observed phenotype is highlighted, and elaborate on the cellular key mechanisms orchestrated by SMAD6 in the development of these three discrete inherited disorders. In addition, we discuss future research needed to elucidate the pathogenetic mechanisms underlying these diseases in order to improve their molecular diagnosis, advance therapeutic strategies and facilitate counselling of patients and their families.

The protein SMAD6, encoded by *SMAD6* (OMIM: 602931), belongs to the SMAD family of proteins involved in the bone morphogenetic proteins (BMP) signalling cascade. Even though these molecules were initially discovered for their ability to induce bone formation, it is now clear that BMPs are important in the embryogenesis and development of many organ systems, as well as in maintenance of adult tissue homoeostasis. SMAD6 is an intracellular inhibitor of, predominantly, the BMP signalling pathway, yet it cross-talks with the closely related transforming growth factor-β (TGF-β) signalling pathway^1,2^.

Over the past 10 years, genetic variants in *SMAD6* were demonstrated to impinge on the risk of human genetic disorders^3–13^ such as cardiovascular diseases, including congenital heart defects (CHD), craniosynostosis (CRS) and radioulnar synostosis (RUS). CHD is among the most common birth defects, affecting 6–13:1000 live-born infants^14–16^. In association with SMAD6-deficiency, it encompasses a range of cardiac and outflow tract abnormalities. Complex lesions consisting of multiple defects are often severe, even critical, for which treatment with advanced surgery for definitive correction of malformations or (palliative) medication is imperative^17^. In addition, adult patients with a sole congenital aortic valve defect associate with more late-onset vascular complications like a pathological widening of the thoracic aorta (~thoracic aortic aneurysm (TAA))^18^. TAAs are also life-threatening as they are (1) clinically silent^19^, (2) entail a high risk for acute dissection and/or rupture (mortality rates ≥70%)^20^, and (3) no therapy currently exists that can stop TAA development or progression^21^. CRS, which is a skull defect afflicting 1:2100–2500 live births^22,23^, is a second *SMAD6*-related disease. Surgical correction is frequently necessary to prevent complications^24^ such as developmental delay, facial abnormality, sensory, respiratory and neurological dysfunction, anomalies affecting the eye, and psychological disturbances^25^. Finally, congenital RUS, also referred to as fused forearm bones, is a rare condition with ~500 cases reported in literature^9,13,26^. This malformation, usually diagnosed before the age of 5 years, is not life-threatening, but corrective surgery and/or medication to control pain might, in some cases, improve the quality of life^13^.

The therapeutic strategies for *SMAD6* mutation-positive patients mainly focus on disease monitoring in order to define the appropriate time for intervention, medication to control pain, and surgical repair^19,24,27–30^. Even though surgery is effective, it is associated with risks, requires early detection of at-risk patients, only provides relief late in the disease course, and does not target the underlying driver(s) of the disease. Hence, there is a need for a better (molecular) understanding for early diagnosis, and to empower new therapies to prevent disease progression. With this review, we provide a comprehensive overview on *SMAD6*-deficiency in human genetic disorders by summarising the clinical, (patho)genetic and cellular (dis)similarities observed in human and mouse models. We conclude with future directions of research needed to improve patient management based on the underlying *SMAD6*-related molecular disease signature.

## Clinical phenotype of patients with SMAD6-deficiency

The clinical presentation of heterozygous *SMAD6* variant-positive patients is extremely heterogeneous as illustrated by the different affected organ systems, the varying degree of severity, and the range of associated complications. Table 1 summarises the clinical phenotype of probands with disease-causative *SMAD6* variants. All disease-related clinical definitions are summarised in Table 2.

**Table 1:** Clinical phenotype of probands with disease-causative SMAD6 variants.

Study	Patient cohort	Major defect (sub-category)	Number of probands	NS/S	Age	Additional features within the same organ system
Tan et al.^10^	Cardiovascular malformations	Bicuspid aortic valve (LVO)	1	NS	1.5 y^a^	Aortic stenosis
	Cardiovascular malformations	Bicuspid aortic valve with coarctation of the aorta (LVO)	1	NS	30 y^a^	Aortic stenosis
Timberlake et al.^11^; Timberlake et al.^12^	Craniosynostosis	Craniosynostosis	17	NS	Paediatric^b^	Metopic synostosis (*N* = 12), sagittal synostosis (*N* = 3), metopic + sagittal synostosis (*N* = 2)
Jin et al.^5^	Congenital heart defect	Tetralogy of Fallot (CTD)	3	Unknown^c^	Paediatric^b^	Pulmonary stenosis (subvalvular (*N* = 2), valvar (*N* = 1)), ventricular septal defect (malalignment) (*N* = 1), coronary artery anomaly (right) (*N* = 1), left aortic arch with normal branching pattern (*N* = 1), patent foramen ovale (*N* = 1)
	Congenital heart defect	Transposition of the Great Arteries (CTD)	1	Unknown^c^	Paediatric^b^	Atypical coronary arteries in D-loop Transposition of the Great Arteries, transposition D-loop of the Great Arteries with intact ventricular septum, left aortic arch with a normal branching pattern
	Congenital heart defect	Hypoplastic left heart syndrome (LVO)	1	Unknown^c^	Paediatric^b^	Aortic arch hypoplasia, aortic atresia, hypoplasia ascending aorta, hypoplastic left ventricle (subnormal cavity volume), mitral atresia, restrictive patent foramen ovale
	Congenital heart defect	Coarctation of the aorta (LVO)	1	Unknown^c^	Paediatric^b^	Atrial septal defect (secundum), left-sided patent ductus arteriosus, tubular hypoplasia of aorta, ventricular septal defect (malalignment, muscular outlet)
	Congenital heart defect	Bicuspid aortic valve with coarctation of the aorta (LVO)	1	Unknown^c^	Paediatric^b^	Aortic arch hypoplasia, hypoplastic left ventricle (subnormal cavity volume), patent foramen ovale
	Congenital heart defect	Other	1	Unknown^c^	Paediatric^b^	Left aortic arch with normal branching pattern, partially anomalous pulmonary veins, sinus venosus septal defect (superior type)
	Congenital heart defect	Other	1	Unknown^c^	Paediatric^b^	Vascular ring, aberrant left subclavian artery, abnormal branching right aortic arch, right aortic arch left ligament
Gillis et al.^4^; Luyckx et al.^7^; Park et al.^8^	Cardiovascular malformations	Bicuspid aortic valve-related thoracic aortic aneurysm	15	NS	Average 64.1 y	Aortic valve calcification (*N* = 1), coarctation of the aorta (*N* = 2), aortic regurgitation (*N* = 1), aortic stenosis (*N* = 1)
Kloth et al.^6^^d^	Cardiovascular malformations	Coarctation of the aorta (LVO)	1	S^e^	6 y	Suspected tricuspid aortic valve
		Other	1	NS	10 y	Dysplastic and stenotic pulmonary valve, dilated cardiomyopathy, stenotic left main coronary artery
Yang et al.^13^; Shen et al.^9^	Radioulnar synostosis	Radioulnar synostosis	77	NS	Average 5 y	Lateral left radioulnar synostosis (*N* = 17), lateral right radioulnar synostosis (*N* = 6), bilateral radioulnar synostosis (*N* = 54)
Calpena et al.^3^	Craniosynostosis	Craniosynostosis	26	S^f^/NS^g^	Not reported	Metopic synostosis (*N* = 15), sagittal synostosis (*N* = 6), right coronal synostosis (*N* = 2), sagittal + left coronal synostosis (*N* = 1), sagittal + bicoronal synostosis (*N* = 2)

*NS* non-syndromic, *S* syndromic, *LVO* left ventricular obstruction, *CTD* conotruncal defect, *y* years, *NA* not applicable.

This table excludes patients with known disease-related genetic hits at other loci in addition to *SMAD6*.

^a^Unclear if the aorta has been evaluated.

^b^Age is not specified.

^c^Two patients had extra-cardiac abnormalities (i.e., syndromic cases).

^d^Only paper describing bi-allelic variants.

^e^Consanguineous family with facial dysmorphism, unilateral hypoplasia, bilateral radioulnar synostosis, bilateral toe 2/3 syndactyly, very dry and scaly skin, dysrhythmic electro-encephalogram without seizure activity and mild intellectual disability.

^f^Seven syndromic probands.

^g^Nineteen non-syndromic probands.

**Table 2:** Clinical description of the disease-related anomalies.

Anomalies	Clinical description
Absent corpus callosum	A congenital brain defect with partial or complete absence of the region that connects the two cerebral hemispheres.
Atrial septal defect	A congenital heart defect resulting from incomplete atrial septation.
Atrioventricular septal defect	A congenital heart defect resulting from incomplete septation of the atrioventricular canal.
Bicuspid aortic valve	A congenital heart defect in which the aortic valve has only two leaflets instead of the normal three.
Caudal vertebrae dysplasia	A congenital defect of a total or partial failure of the development of the caudal vertebrae.
Coarctation of the aorta	A congenital heart defect in which blood flow is blocked by aortic narrowing usually at the region of the ductus arteriosus.
Coronal synostosis	A congenital skull defect in which the coronal suture close prematurely leading to flattening of the head (unicoronal), or a short head with wide appearance (bicoronal).
Dilated cardiomyopathy	A condition in which the heart becomes enlarged and cannot pump blood effectively.
D-loop	Refers to the normal rightward (dextro = D) loop or bend of the embryonic heart tube and indicates that the inflow portion of the right ventricle is to the right of the morphological left ventricle.
Frontal bossing	A condition indicating a protuberance of the frontal bones of the forehead.
Hypoplastic left heart syndrome	A congenital heart defect in which the heart’s left side (including the aorta, aortic valve, left ventricle and mitral valve) is underdeveloped.
Macrocephaly	A condition in which circumference of the head is more than two standard deviations above the mean value for a given age and gender.
Metopic synostosis	A congenital skull defect in which the metopic suture close prematurely leading to a forehead with triangular appearance (trigonocephaly).
Microcephaly	A condition in which circumference of the head is more than two standard deviations below the mean value for a given age and gender.
Mitral/pulmonary/tricuspid/aortic valve regurgitation	A condition in which the valve does not close properly, allowing blood to flow backwards. Regurgitation is also called insufficiency or incompetence.
Patent ductus arteriosus	A congenital heart defect in which the ductus arteriosus fails to close after birth.
Patent foramen ovale	A congenital heart defect in which the foramen ovale did not close properly at birth, with the result of an existing hole between the left and right atria of the heart.
Plagiocephaly	A condition in which the skull flattens on one side.
Polydactyly	A congenital skeletal condition in which an individual has more than 5 fingers per hand or 5 toes per foot.
Premature fusion of the anterior fontanel	A congenital skull defect in which the anterior fontanel close prematurely.
Radioulnar synostosis	A congenital defect in which the radius and ulna of the forearm is abnormally connected (synostosis).
Sagittal synostosis	A congenital skull defect in which the sagittal suture close prematurely leading to a long and narrow head (scaphocephaly).
Sinus venosus septal defect	A congenital heart defect in which a deficiency of the common wall between the superior vena cava and the right upper pulmonary vein is present thereby allowing shunting of blood from the systemic to the pulmonary circulation.
Stenotic left main coronary artery	A condition in which the left main coronary artery is narrowed.
Stenotic pulmonary valve	A condition in which the pulmonary valve is narrowed.
Tetralogy of Fallot	A congenital heart defect characterised by right ventricular outflow tract obstruction, right ventricular hypertrophy, ventricular septal defect and overriding aorta.
Thoracic aortic aneurysm	A condition in which the aortic diameter is more than two standard deviations above the mean value for a given age and gender.
Transposition of the Great Arteries	A congenital heart defect referring to ventriculoarterial discordance, i.e., aorta arises from a morphological right ventricle, and the pulmonary artery arises from a morphological left ventricle.
Vascular ring	A congenital heart defect in which the aorta or its branches forms a ring around the trachea and the oesophagus.
Ventricular septal defect	A congenital heart defect resulting from incomplete ventricular septation.
Ventriculomegaly	A condition in which the brain ventricles are abnormally enlarged.

### Cardiovascular diseases

The cardiovascular phenotype (cases, *N* = 31) (probands, *N* = 28, Table 1) include left ventricular outflow tract defects (*N* = 21/28, 75%)^4,5,7,8,10^, conotruncal defects (*N* = 4/28, 14%)^5^, and defects defined as “others” (*N* = 3/28, 11%)^5^ as they cannot be categorised into the traditional subgroups that arise from disruption of shared embryonic processes. Left ventricular outflow tract defect refers to hypoplastic left heart syndrome (HLHS) (*N* = 1/21, 5%), coarctation of the aorta (CoA) (*N* = 2/21, 10%), and bicuspid aortic valve (BAV), which is associated with congenital CoA and/or late-onset TAA (N = 17/21, 81%) in all patients, except for one toddler with isolated BAV (*N* = 1/21, 5%; 1.5 years old). Conotruncal defects include Tetralogy of Fallot (*N* = 3/4, 75%) and D-loop transposition of the Great Arteries (*N* = 1/4, 25%). The remaining three patients presented with either a vascular ring, partially anomalous pulmonary veins combined with sinus venosus septal defect, or stenotic pulmonary valve and stenotic left main coronary artery accompanied with dilated cardiomyopathy.

### Craniosynostosis

The clinical outcome of CRS (cases, *N* = 49) (probands, *N* = 43, Table 1)^3,11,12^ involves syndromic (*N* = 7/43, 16%) and non-syndromic presentations (*N* = 36/43, 84%) in which single and multiple fusion events of almost all sutures have been identified. Most common presentation was metopic synostosis (*N* = 27/43, 63%), followed by sagittal synostosis (*N* = 9/43, 21%), right unicoronal synostosis (*N* = 2/43, 5%), combined metopic and sagittal synostosis (*N* = 2/43, 5%), combined sagittal and bicoronal synostosis (N = 2/46, 4%), and combined sagittal and left unicoronal synostosis (*N* = 1/43, 2%). Remarkably, raised intracranial pressure following cranial reconstruction, which is usually a rather infrequent complication in simple synostosis of midline sutures, should be specifically monitored in *SMAD6* variant*-*positive patients^3^. Other recurrent brain or skull anomalies in (non-)syndromic subjects comprise ventriculomegaly and absent corpus callosum, macrocephaly, and mild microcephaly, and mild-to-moderate neurodevelopmental delay (consisting of speech, educational and global delay)^3,11,12^. More subtle learning difficulties were observed in 36% of the non-syndromic patients (*N* = 14)^11^, while gross and fine motor delays were only observed occasionally^3,11^. In syndromic cases^3^, cardiac defects were common (*N* = 5/7, 71%), but seem to have another pattern which is further discussed in the following section on the clinical overlap.

### Congenital radioulnar synostosis

Patients with RUS^9,13^ (cases, *N* = 93) (probands, *N* = 77, Table 1) are most frequently characterised by bilateral RUS (69%; isolated (*N* = 42/61), familial (*N* = 22/32)), followed by unilateral left-sided RUS in sporadic patients (*N* = 15/42, 36%) versus right-sided RUS (*N* = 4/42, 10%), while no susceptibility for left- or right-sided RUS was observed in families (*N* = 5/32, 16%). Affected individuals within a single pedigree can show both bilateral and unilateral RUS. No syndromic cases have been reported thus far, yet some subordinate clinical findings have been described in 14 families^9,13^: three families presented with axial skeletal deformities (including cervical fusion, rib malformation, caudal vertebrae dysplasia and vertebral malformations), two families had polydactyly (pre- and pro-axial type), five families exhibited CHD (encompassing patent ductus arteriosus, mitral/tricuspid/aortic regurgitation, atrial septal defect, BAV, left ventricular hypertrophy and mitral/pulmonary valve insufficiency), and four families showed skull-related defects (containing frontal bossing, plagiocephaly and premature closure of anterior fontanel, but no unequivocal description of CRS). Remarkably, in six out of the 14 families a variant-positive *SMAD6* carrier without RUS but with skeleton-, skull-, or CHD-related features^9,13^ was reported: two affected individuals from two families displayed cervical fusion or caudal vertebrae dysplasia, two patients from two families exhibited with premature closure of anterior fontanel, frontal bossing and plagiocephaly or solely plagiocephaly, one subject presented with polydactyly and, finally, one affected had patent ductus arteriosus together with mitral regurgitation.

### Clinical overlap?

Studies have, in addition to the clinical indication for study enrolment, to some extent also assessed the presence of other *SMAD6*-related clinical associations. Patients with cardiovascular disease did not exhibit CRS and/or RUS. A child, from a consanguineous family harbouring a pathogenic homozygous *SMAD6* variant, was reported to present with CoA, suspected tricuspid aortic valve, bilateral RUS, renal anomalies, facial dysmorphism and global development delay^6^. In view of consanguinity, homozygosity at other loci as an explanation for these multisystemic features cannot be excluded. CRS cases did not exhibit RUS, but five syndromic CRS cases presented with a CHD^3^ of which none seem to mimic the more severe conotruncal and outflow tract defects seen in the cardiovascular disease cohorts. For example, defects in three patients resolved spontaneously. One patient with atrioventricular septal defect required surgery at the age of three years, yet his variant-positive mother had a normal echocardiogram. The fifth patient had BAV (*N* = 1/46, 2%) with right bundle branch block. This observation exactly matches the epidemiological number of 2% for BAV in the general population though^31^. None of the seven extra screened asymptomatic parents of *SMAD6* variant-positive children with CRS showed any evidence for BAV or TAA^3^. Finally, no BAV or TAA has been identified so far in the non-syndromic CRS cohort (personal communication with A. Wilkie, Oxford). Hence, no clinical overlap of a variant-positive *SMAD6* carrier with cardiovascular disease or CRS with any abnormality affecting the other organ systems has been observed to date.

Finally, the phenotypic picture in 14 families with RUS is more complicated as both CHD as well as skull and skeletal abnormalities have been observed occasionally (*N* = 12/93, 13%)^9,13^. Although based on their nature and incidence, we cannot rule out an alternative cause for some abnormalities (e.g., valve insufficiency, left ventricular hypertrophy and rib/vertebral malformation), the occurrence of skeletal- (*N* = 4/93, 4%), skull- (*N* = 9/93, 10%), or CHD-related (*N* = 3/93, 3%) abnormalities in families with RUS does hint to some clinical overlap. For example, two variant-positive *SMAD6* carriers from two families without RUS presented with axial skeletal deformities, either cervical fusion or caudal vertebrae dysplasia. Extra skull features were observed in another five families, including frontal bossing (*N* = 4/93, 4%), plagiocephaly (*N* = 3/93, 3%), and premature fusion of the anterior fontanel (*N* = 2/93, 2%). Plagiocephaly and premature fusion of the anterior fontanel was reported in a variant-positive family member without RUS. And finally, three families had CHD too, namely patent ductus arteriosus (*N* = 1/93, 1%), atrial septal defect (*N* = 1/93, 1%), and BAV (*N* = 1/93, 1%).

## Genetic (dis)similarities between *SMAD6*-related disorders

**Fig. 1:**
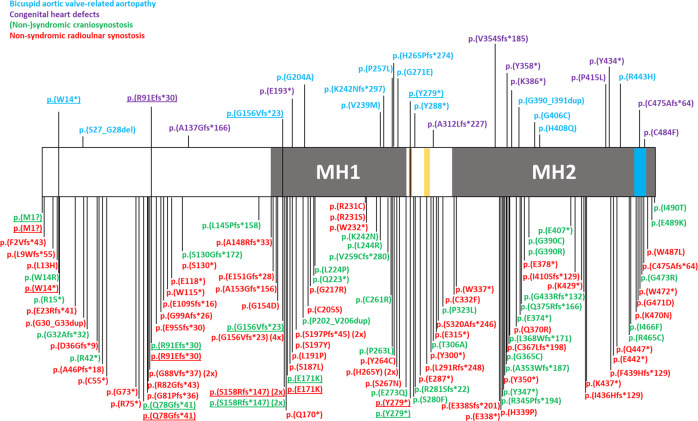
Graphical representation of identified heterozygous SMAD6 variants in probands with bicuspid aortic valve-related aortopathy, congenital heart disease, (non-)syndromic craniosynostosis and non-syndromic radioulnar synostosis. SMAD6 protein has several domains: MH1-domain (grey; inhibitory effect on signalling), PY motif (orange), PLDLDS motif (yellow), MH2-domain (grey; inhibitory effect on signalling) and L3-loop (blue; determines specificity for interaction with type I receptors)^88,89^. Of note: (1) all variants were called “pathogenic” in the original publications but there is different criteria for calling “pathogenicity” (e.g., by applying different allele frequency thresholds, and/or using specific functional tests); (2) identical protein changes (underlined) are described in patients with distinctive phenotypes.

Intriguingly, similar, or even identical, heterozygous loss-of-function variants in *SMAD6* cause these three distinct disorders (Fig. 1 and Supplementary Table 1)^3–13^. The variant spectrum includes rare truncating and missense variants locating in the functional MH1- and MH2-domain of the protein with no phenotypic correlation with respect to variant type nor location. Identical nucleotide changes (*N* = 6) have been described in patients with cardiovascular disease (*N* = 3), CRS (*N* = 5) or RUS (*N* = 10). Moreover, the phenotype within these families are, predominantly, restricted to one affected organ system. For example, the p.(Gly156Valfs*23) variant causes BAV-related aortopathy (*N* = 1), sagittal synostosis (*N* = 1), and non-syndromic RUS (*N* = 4), for which no clinical overlap has been documented except for frontal bossing in one family with left-sided RUS. Hence, the molecular finding cannot predict the clinical presentation of a patient, and, as such, it is likely that (a) factor(s) inherited together with the primary *SMAD6* mutation drives the resultant patient phenotype. The latter seems likely as within one family concordance of the phenotype is frequently observed.

### Cardiovascular disease

The aetiology of CHD is multifactorial, involving genetic and environmental factors such as smoking, alcohol abuse and infection transmitted by the mother during pregnancy^32^. Familial studies have demonstrated that the CHD recurrence risk in family members of affected individuals depends on the type of lesion^33^. Pathogenic variants cause autosomal dominant, autosomal recessive, or X-linked traits with variable penetrance and clinical expressivity. About 132 definitive and strong candidate genes for CHD in numerous functional classes like chromatin modification, transcription factors and signal transduction, amongst others, have been reported. The predominant disease-causative effect is through loss-of-function^34^. To date, 50% of the patients remain molecularly undiagnosed though, and the yield is even lower in non-syndromic cases^34^. Interestingly, pathogenic *SMAD6* variants have been shown to be enriched in isolated paediatric and adult CHD patients, in which most patients exhibited left ventricular outflow tract defects (Table 3). So far, patients with recessive variants do not seem to present with a more severe cardiovascular phenotype as compared to subjects harbouring heterozygous variants. However, this observation is based on only two cases, and no functional analyses have been performed^6^. A *SMAD6* genetic uptake of 4.6% was reached in more severely affected BAV-related aortopathy patients, i.e., BAV patients who underwent surgical repair for aneurysmal disease before the age of 50, and with a positive family history for cardiovascular disease. The estimated penetrance for the disease was 82.4%. *SMAD6* is the most important BAV/TAA gene identified thus far, as none of the approximately 30 definitive and candidate genes for BAV and/or TAA explain more than 1% of these patients^7^. The emerging BAV/TAA disease-related pathways include impaired cardiac transcription factor activity (e.g., *GATA5*)^35,36^, perturbed extracellular matrix homoeostasis (e.g., *LOX*)^37^, aberrant TGF-β (e.g., *TGFBR1*)^19^ and NOTCH (e.g., *NOTCH1*)^38^ signalling, deficiency of the vascular smooth muscle cell contractile apparatus (e.g., *ACTA2*)^39^, and altered endothelial cell function (e.g., *ROBO4*)^40^. Taken together, carrying a pathogenic *SMAD6* might be insufficient to definitively cause cardiovascular disease in all cases, and, as such, more research is required to identify the missing information, and to understand how it contributes to disease.

**Table 3:** SMAD6 variant-positive patients with congenital heart disease.

	Tan et al.^10^	Jin et al.^5^	Gillis et al.^4^	Luyckx et al.^7^	Total
Left ventricular outflow tract	2/83 (2.4%)	3/797 (0.4%)	11/441 (2.5%)	3/65 (4.6%)	19/1386 (1.4%)
D-loop transposition of the Great Arteries	0/65 (0%)	1/251 (0.4%)	–	–	1/316 (0.3%)
Conotruncal defects	0/78 (0%)	3/872 (0.3%)	–	–	3/950 (0.3%)
Heterotaxy	0/10 (0%)	0/272 (0%)	–	–	0/282 (0%)
Others	0/200 (0%)	2/679 (0.3%)	–	–	2/879 (0.2%)
Total	2/436 (0.5%)	9/2871 (0.3%)	11/441 (2.5%)	3/65 (4.6%)	

This table excludes (1) patients with known disease-related genetic hits at other loci, in addition to *SMAD6*, and (2) case reports lacking information on the total number of screened patients.

A clinical and genetic association between BAV, HLHS, and CoA have already been thoroughly discussed in familial studies^41,42^, and some examples of monozygotic twins with discordant phenotypes, i.e., one has BAV while the other present with HLHS, have been described^43,44^. As SMAD6-deficiency results in a spectrum of, mainly, left ventricular outflow tract defects, one could hypothesise the existence of additional genetic hits in families. Particular emphasis might be given to ascertain essential cardiac transcription complexes, and to investigate the accessibility of these factors onto DNA in patient-derived material in order to reveal novel crucial clues on the pathogenesis of CHD disease.

### Craniosynostosis

CRS is a heterogeneous disease influenced by mechanical and extrinsic forces as well as genetic components affecting the intrinsic properties of the suture^45^. In families, an autosomal dominant mode of transmission is mostly observed, but in about half of the cases a de novo variant is found. The genetic uptake is highest in syndromic cases, while isolated cases (i.e., 75% of all patients) largely remain molecularly undiagnosed^45,46^. Approximately one quarter of CRS cases harbour a disease-causative variant in one of the known genes, mostly in *FGFR2*, *FGFR3*, *TCF12, ERF, EFNB1*, or *TWIST1*, causing either a loss- or gain-of-function. These gene products are involved in signal transduction pathways like FGF signalling (*FGR2, FGFR3*), Eph/ephrin signalling (*EFNB1*) and ERK-MAPK activity (*ERF*) or they bind DNA to regulate gene expression (*TWIST1, TCF12*). As *SMAD6* variants account for 5.8% of all (non-)syndromic patients with metopic synostosis, it became, by far, the largest monogenic contributor to metopic synostosis yet identified. Furthermore, *SMAD6* variants seem less commonly associated with other types of suture fusion^3^, making it in particular relevant to screen patients with metopic synostosis for *SMAD6* deficiency.

*SMAD6*-related CRS has been associated with reduced penetrance (overall penetrance, 16–24%)^3,11,12^. As such, a two-locus inheritance model for CRS (i.e., metopic, sagittal and combined metopic and sagittal) was proposed by Timberlake et al., in which near complete-penetrance was reached upon co-occurrence with a common *BMP2* SNP risk allele (C) (rs1884302)^11^. Upon merger of datasets, this association still holds true, yet the initial signal has weakened due to non-replication in an independent cohort. One explanation might be the underrepresentation of the risk allele (frequency ~0.33, gnomAD: European non-Finnish) in non-penetrant *SMAD6* variant harbouring individuals in the discovery studies, which was not observed in a third study^3,11,12^. Additionally, rs1884302 was found to strongly associate with sagittal synostosis^47^, and more recent data for metopic synostosis reveal no equivalent association for this SNP^48^. Extra work is necessary to explore on such relationship between *SMAD6* variant-positive patients with sagittal synostosis, and a larger sample size is needed to dissect whether this interaction is truly digenic inheritance or is merely an additive effect of the GWAS signal, modifying the penetrance of *SMAD6* pathogenic variants. Additional light was shed onto this digenic inheritance model by revealing the presence of this common SNP in *SMAD6* mutation-positive patients with either BAV-related aortopathy (*N* = 4)^7^ or radioulnar synostosis (*N* = 7)^13^ but in the absence of any sign of CRS. Altogether, current data suggest that the pathogenic *SMAD6* variant alone might be insufficient to definitively cause CRS in all cases, and it still remains to be further investigated what the extra hits, and what the underlying mechanisms are.

### Radioulnar synostosis

Since the 70’s, congenital RUS is recognised as an inheritable disease segregating in an autosomal dominant manner^49,50^. In total, 10% of the RUS patients were identified with a monogenetic cause (e.g., *NOG*) or with aneuploidy syndromes, in which the syndromic subjects presented with additional abnormalities in the skeleton, heart, urinary tract, blood and males had extra X and Y chromosomes^51^. At present, SMAD6 deficiency is, by far, the most important known disease gene for non-syndromic RUS, as it explains 42% of familial cases and 16% of sporadic patients^9,13^. The penetrance of disease is incomplete, and has been reported around 20–25%^9,13^. Other genetic causes include two pathogenic variants in *NOG*, explaining less than 1% of the patients^13^. *NOG* encodes noggin, which is a major BMP antagonist. Dysregulation of BMP signalling due to NOG deficiency in mice showed interference with hedgehog signalling for BMP-induced interdigital cell death^52^, and for axial skeleton development^53^. The contribution of genetic variability in *SMAD6* and *NOG* to syndromic RUS is yet unexplored. In sum, literature indicates that radioulnar synostosis is not exclusively caused by one pathogenic *SMAD6* variant in all cases. Again, more investigation is needed to fill our gap in knowledge about the extra hits and underlying mechanisms.

### Current challenges in SMAD6-related diagnosis and counselling

Patient management for *SMAD6*-related disorders is challenging as rare pathogenic loss-of-function variants associate with (1) reduced penetrance, (2) extreme variability in phenotypical expression, and (3) distinctive clinical entities without genotype–phenotype correlation, as outlined above. Hence, every single case should be discussed thoroughly in a multidisciplinary team based on phenotype, family history, inheritance pattern, and pathogenicity of the variant. Given the possibility of a devastating cardiovascular outcome, echocardiographic evaluation is currently indicated in a *SMAD6* variant-positive proband, irrespective of the clinical indication for referral. A genetic test is best offered to family members of *SMAD6* variant-positive patients with cardiovascular disease or RUS as some clinical overlap with the cardiovascular disease might exist. In contrast, variant-positive *SMAD6* carriers in CRS cohorts are frequently unaffected making a genetic test uninformative. There is currently some preliminary evidence that phenotypes are quite consistent in a single family. Nevertheless, more insight is needed before we can abandon echocardiographic evaluation in relatives of *SMAD6* variant-positive probands with CRS. Another counselling challenge is caused by the observation that the general population well-tolerates loss-of-function *SMAD6* variants (pLI = 0, gnomAD v2.1.1), despite the overwhelming overrepresentation of such variants in disease cohorts as compared to this control population^3–5,11–13^. This is in particular challenging for CRS given the low penetrance of CRS in individuals heterozygous for pathogenic *SMAD6* variants^3,11^.

Lastly, diagnostic and research laboratories also encounter difficulties for variant interpretation, in particular for missense variants. In this regard, Calpena et al.^3^ have provided a filtering strategy able to discriminate high-penetrant rare pathogenic missense variants, as proven in functional tests assessing protein stability and/or impaired BMP signalling activity. Even though very useful, this approach will not classify all type of variants (e.g., 5′ untranslated region), and current bio-informatic tools are not sufficient sensitive to assess variants with moderate effects, which are likely to explain, to some extent, the variability in expressivity and unpredictable penetrance. Nonetheless, implementation of flexible, preferably high-throughput, functional assays for variant interpretation, combined with further refinement of bio-informatic tools, is necessary to address this challenge.

## Lessons from mouse models

Genetically modified mouse models have, with success, been used to interrogate the pathomechanisms underlying rare human disorders. At present, three mouse models lacking the murine orthologue of SMAD6, i.e., Madh6, have been studied (Table 4). The Madh6-mutant mice were produced by a LacZ/neomycin resistance cassette inserted into the 5´ terminus of the exon encoding the MH2-domain of Smad6^54^. Each model is unique by its respective genetic background as all models were generated using embryonic stem cells created by Galvin et al.

**Table 4:** Overview of the published Madh6 knock-out mouse models with the phenotypic characterisation of Madh6^−/−^ animals.

	Galvin et al.^54^	Estrada et al.^55^	Wylie et al.^56^
Generation model	Embryonic stem cells with transgene interrupting SMAD6 function (i.e., insertion of LacZ/neomycin resistance cassette into the 5´ terminus of the exon encoding the MH2-domain)	Stem cells from Galvin et al.	Stem cells from Galvin et al.
Biological consequence	Madh6-LacZ fused transcript	See Galvin et al.^54^	See Galvin et al.^54^
Parents of breeding	Heterozygous	Heterozygous	Heterozygous
Lethality	Partial lethality of madh6^−/−^ mice (P21; 3–13% ~genetic background^a^)	Lethality of madh6^−/−^ mice (P0; 5%, but all died <24 h)	Lethality of madh6^−/−^ mice (P0; 8%, but all died within 2–6 days of birth)
Cardiac phenotype	Hyperplasia cardiac valves, enlarged mitral valve, enlarged pulmonary valve, abnormal truncus arteriosus septation^b^, aortic ossification^c^, hypertension	Not investigated	No hyperplastic valves, or other major defects explaining cause of death
Vascular phenotype	Decreased vasodilation^c^, abnormal thrombosis^d^	Not investigated	Blood vessel haemorrhages in skin and brown fat pads
Craniofacial phenotype	Not observed	Domed skull, shortened snout	Nothing obvious that could explain the cause of death
Axial skeletal phenotype	Not observed	Posterior transformation of cervical vertebrae, bilateral ossification centres in lumbar vertebrae, bifid sternebrae	Nothing obvious that could explain the cause of death
Appendicular skeletal phenotype	Not observed	Smaller size, abnormal growth plate development^e^	Nothing obvious that could explain the cause of death
Genetic background	129/SvEv × BALB/cBy, 129/SvEv × C57Bl/6, inbred 129/SvEv	C57BL/6J x BALB/c	CD1

Madh6 is the murine orthologue of human SMAD6.

^a^Background sensitivity: inbred 129S/SvEv (3% versus expected 25%), mixed 129S6/SvEvTac × BALB/cByJ (9% versus expected 25%), mixed 129S6/SvEvTac × C57BL6/J (13% versus expected 25%).

^b^A subset of homozygotes exhibit abnormal septation of the outflow tract leading to a severely narrowed ascending aorta, and an enlarged pulmonary trunk or the reverse.

^c^Only observed in the surviving animals starting at 6 weeks of age.

^d^Surviving homozygotes display occasional thrombotic lesions as well as focal ischaemia in the lung, liver and kidney.

^e^Abnormal growth plate development: delayed onset of hypertrophic differentiation and mineralisation at midgestation, but expanded hypertrophic zone at late gestation.

In the model on a mixed 129/SvEv × BALB/cBy background^54^, homozygous animals exhibited hyperplasia of the cardiac valves, with the mitral and pulmonary valve being more extremely affected, septation defects, and lethality. The latter was observed due to an underrepresentation of homozygotes at the time of weaning. Surviving animals developed aortic ossification with notable cartilaginous metaplasia and trabeculation of the aortic media (from 6 weeks of age), decreased vasodilation and hypertension. Subsequent in-depth characterisation revealed an excess of mesenchymal cells in the cardiac valves in all homozygotes, while the following was only observed in a subset of the animals: (1) abnormal septation of the outflow tract, i.e., a severely narrowed ascending aorta and an enlarged pulmonary trunk or the reverse, (2) thrombotic lesions and ischaemia in lung, liver, and kidney, (3) subepicardial vascular malformations in the ventricular wall with loss of multiple smooth muscle cell layers in large vessels, and (4) thickening of the endocardium. Interestingly, a background sensitivity for the survival of homozygotes up to weaning was observed by comparing mouse models on different genetic backgrounds (i.e., 129/SvEv × BALB/cBy, 129/SvEv × C57Bl/6, inbred 129/SvEv), which corresponded to the severity of cardiac defects. Heterozygotes were not further studied, and no gender-specific analyses were performed. Even though similar anomalies were described in humans, it is still unanswered whether these mice also present BAV, aortic valve calcification, hypoplastic left heart and what the relative position of the aorta and pulmonary artery is. No gross non-cardiovascular anomalies were described, yet this has not been investigated into detail.

The next-studied knock-out mouse model^55^, on a C57BL/6J × BALB/c background, was generated to investigate the consequences of Smad6 loss during cartilage development. Homozygotes displayed craniofacial anomalies like a domed skull and shortened snout, but no defects in cranial sutures were found. Abnormalities in the skeleton were observed too, such as posterior transformation of cervical vertebrae (C7), flatter thoracic vertebral bodies, presence of bilateral ossification centres in lumbar vertebrae, and bifid sternebrae due to incomplete sternal band fusion. In addition, homozygotes were smaller in size, as confirmed by shorter appendicular bones, and stage-specific defects in endochondral bone formation were found like the delayed onset of hypertrophy at midgestation and expanded hypertophic zone at late gestation. Furthermore, significant embryonic and neonatal lethality was observed, as merely 5% of the progeny were homozygous and all live-born pups died within 24 h after birth due to an unspecified cause. Heterozygotes were not examined in this model, alike with other organ systems, especially no data on the cardiovascular system in the homozygotes were reported.

The last published model^56^ was generated on a CD1 background to elucidate the effects of Smad6 loss on blood vessel development. Wylie et al. reported on embryonic and postnatal lethality of homozygotes (all died by P2–6), in addition to regions of haemorrhages in skin and brown fat pads without any sign of hyperplastic valve thickening in these animals. The observed vessel phenotype was a consequence of disrupted endothelial cell junctions, thereby compromising vessel wall integrity. No in-depth experiments were performed on heterozygous animals, nor other organ systems were examined.

Altogether, this mouse knock-out data support a role for unique genetic background-related clinical presentations. Additional gene expression or pathway analyses in the different Madh6-deficient mouse models might provide essential insights into the pathogenesis of these phenotypes. With respect to the observed cardiovascular phenotype in 129/SvEv × BALB/cBy, 129/SvEv × C57Bl/6 and inbred 129/SvEv Madh6^−/−^ mice, a major codominant modifier gene for lethality might be present. Alike to Tgfβ1^−/−^ mice created on different genetic backgrounds to study angiogenesis^57–59^, independent but epistatically interacting genetic loci might be found that determine the incidence of lethality depending on the model. Interesting modifying genes have already been described to alter the response of lack to TGF-β1 in mice, suggesting that proper TGF-β signalling is key for embryonic survival. Whether by analogy, improper BMP or TGF-β signalling explains the incidence of lethality in Madh6^−/−^ mice with a cardiovascular phenotype remains to be determined.

## Cellular mechanisms orchestrated by SMAD6

Epithelial-to-mesenchymal transition (EMT) is a reversible fundamental biological process for (1) the formation of the body plan, (2) the differentiation of multiple tissues and organs, and (3) to repair tissues. EMT is an extremely coordinated multifaceted process, in which cells disrupt their intercellular adhesion complexes and lose their apicobasal polarity in order to migrate^60,61^. Two highly conserved and critical regulators of EMT are the TGF-β and BMP signalling pathway, which either stimulates or tempers this process, respectively^62^. Hence, SMAD6 modulates EMT by interfering with, predominantly, BMP signalling^54,63^.

**Fig. 2:**
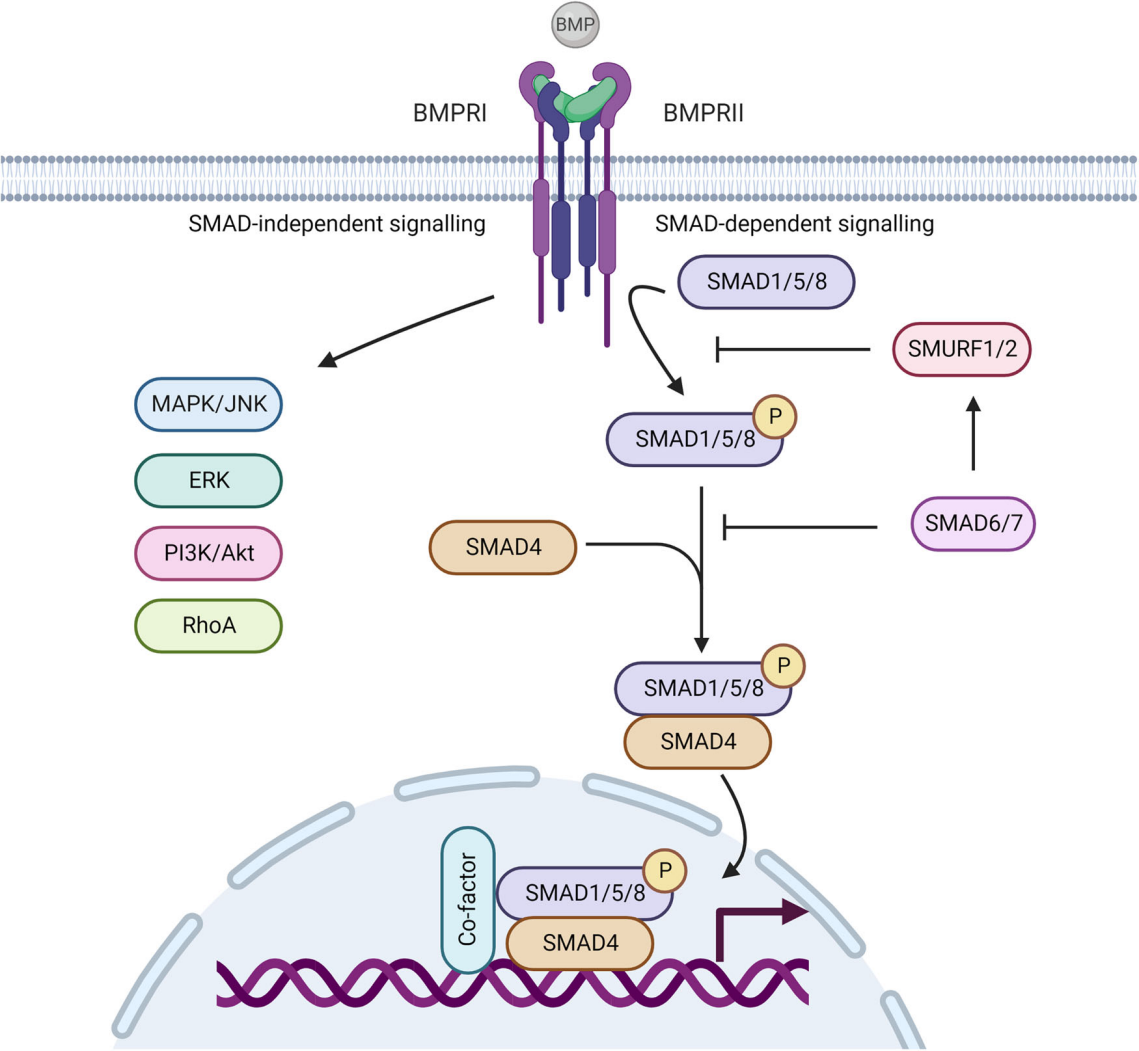
Schematic overview of SMAD-(in)dependent bone morphogenetic protein (BMP) signalling pathway (oversimplification). Upon BMP ligand binding, specific type I and type II receptors form a heterotetrameric complex. The type II receptor phosphorylates the type I receptor, which, in turn, phosphorylates Smad1, Smad5, and Smad8 (canonical BMP signalling). Phosphorylated Smads propagate the signal via complex formation with Smad4 and translocates into the nucleus, where it regulates the expression of BMP-responsive target genes. In addition to Smad activation, activated BMP receptor complexes initiates several intracellular pathways to modulate BMP-dependent cellular responses like PI3-kinase, ERK, RhoA, and MAPK/JNK. Canonical BMP signalling is intracellularly inhibited by inhibitory Smads (Smad6, Smad7) and E3 ubiquitin ligases like Smurf1 and Smurf2. Created with BioRender.com. The figure was exported under a paid subscription.

The mechanosensitive BMP signalling pathway (Fig. 2) regulates cellular lineage commitment, morphogenesis, differentiation, proliferation and apoptosis^64,65^. BMPs activate numerous pathways, of which the SMAD signalling pathway has best been studied^66^. BMP signalling interferes with its own signalling as SMAD6, a direct BMP target, selectively recruits SMURF1 to BMP type 1 receptors^67^ or competes with receptor-regulated Smads for binding to SMAD4^68^, thereby establishing a negative feedback loop. A further level of control is achieved by cross-talk with TGF-β, FGF, MAPK, Hedgehog, PI3K/Akt, Wnt/beta-catenin, retinoic acid and Notch signalling pathways in order to regulate cellular BMP-related processes in a very tight spatial and temporal manner^64,65,69^.

### SMAD6 signalling in cardiovascular development

Dysregulation of BMP signalling has extensively been investigated in numerous cardiovascular diseases^1,70^. Interestingly, SMAD6-deficient patients mainly exhibit defects related to two discrete cell lineages, namely second heart field and neural crest cells. Second heart field cells are multipotent progenitors originating from cardiac progenitor cells and contribute to distinct regions of the myocardium, cardiac endothelial cells and smooth muscle cells^71^, while neural crest cells are derived from the dorsal aorta and migrate as multipotent cells into the developing outflow tract to coordinate outflow tract septation^72^. During cardiac cushion development, SMAD6 is specifically expressed in endothelial cells where it functions in (1) maintaining endothelial to mesenchymal transition (EndMT)^54,63^, (2) stimulating cardiac cushions to grow^73^ and (3) interacting with cardiac neural crest cells^74^, cells required for aorticopulmonary septum formation. As such, this might explain the marked clinical variability of SMAD6-deficient patient with BAV-related aortopathy as predominant phenotype, and, emphasises the complexity of CHD, in which gene dosage, timing, haemodynamic flow, and its interplay with other signalling pathways like Notch and TGF-β are important too. For example, endothelial cells can undergo EndMT to become either myofibroblast-like^75^ or chrondrocyte- and osteoblast-like cells^76^, depending on their cellular context.

### SMAD6 signalling in cranial suture development

Gene discovery studies, and their subsequent characterisation in mice, have determined highly conserved molecular pathways and specific biological processes at different stages in cranial suture development^45^. Initially, the strongest implication of BMP signalling involvement was shown by BMP type 1 receptor (BMPR1A)^77^, and by its convergence at key transcriptional factors downstream of BMP, i.e., Msx2^78^ and Twist1^79,80^, to regulate cell proliferation, mesenchyme condensation, osteoblast differentiation, and osteogenesis. Subsequent work further supported a role of SMAD-dependent signalling by the identification of causal mutations in *SKI*^81^ and *SMAD3*^82^ in Shprintzen–Goldberg and Loeys–Dietz patients, both conditions associated with CRS. Additional evidence has emerged as SMAD6-deficiency increases the risk for CRS, and in particular for metopic synostosis. In literature, metopic synostosis has already been hypothesised to be the consequence of abnormal maturation of neural crest-derived mesenchymal stem cells via disturbed dynamics of cell identity or migration as a common predisposing factor, and this can now be further investigated^83,84^. Alternatively, processes not involved in cranial suture development but affecting osteogenesis such as osteoblast and osteoclast activity could be impaired too, and lead to CRS.

### SMAD6 signalling in radioulnar development

Studies on BMP signalling in radioulnar development are very scarce. So far, published data on RUS is limited to genetic studies^9,13^ and clinical descriptive reports lacking in-depth functional analyses. Our current knowledge is inferred from studies in axial skeletal development, with molecular pathways like Wnt, Hedgehog, Notch, and FGF signalling pathways, to be highly involved^85^. As RUS is believed to be the result of anomalous differentiation and/or segmentation of the adjacent radius and ulna, it could be true that BMPs lead to impaired mesenchymal stem cell differentiation via Runx2 to promote osteoblast differentiation from mesenchymal precursor cells^86,87^.

## Summary and future outlook

In summary, three distinctive human genetic disorders are caused by SMAD6 deficiency without domain-specific or mutation-type genotype–phenotype correlation making proper patient management difficult. Patients with cardiovascular disease or craniosynostosis do not show any manifestations in the other organ system within relatives of a single family, suggesting that, (an)other factor(s) co-segregating with the primary *SMAD6* variant might explain the resultant phenotype. To further explore this hypothesis, in-depth investigation into the identification of the responsible cell type(s) and their identity, as well as defining the predominant affected signalling cascade(s) driving these disorders, will be fundamental for our knowledge. Cell lineage tracing and spatial gene expression analyses in Madh6-deficient mouse models might unravel important clues to discriminate the afflicted processes leading to cardiovascular disease, craniosynostosis and radioulnar synostosis. Furthermore, a detailed clinical and genetic assessment of additional *SMAD6* variant-positive patients will be needed, and, in particular, ascertain the complete phenotypic picture of families with RUS, in which some clinical overlap with CHD-, skull-, and skeletal-related anomalies might exist.

Other (additional) genetic factor(s) might explain incomplete penetrance and extreme variability in phenotypical expressivity in a patient with SMAD6 deficiency. For example, rare (or common) variants located in a regulatory element of the trans-wild-type *SMAD6* allele, or variants in genes (e.g., *SMAD7*) afflicting expression and/or activity of the BMP and/or the closely related TGF-β signalling activity are interesting avenues for further exploration. It is worthwhile to consider genome-wide association approaches that look into rare “second-hit” variants with large effect size in *SMAD6*-deficient patients in order to add novel information to the puzzle. Although this would aid to understand the molecular basis of disease, the current available number of *SMAD6* mutant patients might not be sufficient to detect (a) signal(s) even when only extreme phenotypes would be selected. Nonetheless, in the upcoming years we will confidently identify the *SMAD6*-related molecular patterns associated with these three distinctive genetic disorders. This will allow us to detect early at-risk individuals and empower new therapies.

### Reporting summary

Further information on research design is available in the Nature Research Reporting Summary linked to this article.

## Supplementary information

Reporting Summary Checklist

Supplementary Table 1

## Data Availability

All data generated or analysed during this study are included in this published article (and its supplementary information file).
